# Impact of Preoperative Image Sharing and Anatomical Suitability on EVAR Outcomes for Ruptured Abdominal Aortic Aneurysm

**DOI:** 10.3400/avd.oa.26-00086

**Published:** 2026-09-19

**Authors:** Yuki Hashimoto, Shinsuke Kikuchi, Yuri Yoshida, Hirofumi Jinno, Noriyuki Yokoyama, Daichi Mizushima, Tsutomu Doita, Takayuki Uramoto, Kazuki Takahashi, Seima Ohira, Keisuke Kamada, Takamitsu Tatsukawa, Naoya Kuriyama, Seiko Tanaka, Akihito Tampo, Motoi Okada, Ami Sugawara, Kenichi Takahoko, Yoshiko Onodera, Atsushi Kurosawa, Hiroshi Makino, Daiki Uchida, Nobuyoshi Azuma

**Affiliations:** 1 Department of Vascular Surgery, Asahikawa Medical University, Asahikawa, Hokkaido, Japan; 2 Department of Cardiovascular Surgery, Sapporo Kosei General Hospital, Sapporo, Hokkaido, Japan; 3 Department of Cardiovascular Surgery, Osaka University Graduate School of Medicine, Suita, Osaka, Japan; 4 Division of Nursing, Asahikawa Medical University Hospital, Asahikawa, Hokkaido, Japan; 5 Department of Emergency Medicine, Asahikawa Medical University, Asahikawa, Hokkaido, Japan; 6 Department of Anesthesiology and Critical Care Medicine, Asahikawa Medical University, Asahikawa, Hokkaido, Japan; 7 Department of Vascular Surgery, Asahikawa City Hospital, Asahikawa, Hokkaido, Japan

**Keywords:** ruptured abdominal aortic aneurysm, endovascular aneurysm repair, anatomical suitability, instructions for use, remote image sharing

## Abstract

**Objectives:**

The influence of anatomical suitability on outcomes after endovascular aneurysm repair (EVAR) for ruptured abdominal aortic aneurysm (rAAA) remains uncertain. This study evaluated the association between adherence to device instructions for use (IFU) and clinical outcomes after EVAR for rAAA.

**Methods:**

We retrospectively reviewed 31 consecutive patients who underwent emergency EVAR for rAAA between August 2016 and June 2023. Anatomical suitability was assessed by retrospective computed tomography, and patients were classified as inside or outside IFU. The influence of preoperative image sharing (JOIN; Allm, Tokyo, Japan) on perioperative workflow was also evaluated, with patients divided into image-sharing and non–image-sharing groups. The primary outcome was reintervention; secondary outcomes included 30-day and overall mortality.

**Results:**

Twenty patients (64.5%) were inside IFU and eleven (35.5%) were outside IFU. Outside-IFU cases had shorter proximal neck length and greater neck angulation. Reintervention was more frequent outside IFU than inside IFU (63.6% vs. 10.0%, p <0.01), whereas 30-day mortality (18.2% vs. 10.0%, p = 0.601) and overall mortality (36.4% vs. 60.0%, p = 0.300) were not significantly different. Prehospital image sharing using the JOIN application was associated with shorter door-to-operating room time despite longer transfer distance.

**Conclusions:**

EVAR for rAAA may achieve acceptable early survival in selected outside-IFU cases; however, outside-IFU anatomy is associated with increased reintervention. Careful patient-specific selection and structured follow-up are essential.

## Introduction

Ruptured abdominal aortic aneurysm (rAAA) remains a life-threatening condition with persistently high mortality, and rapid diagnosis and intervention are essential for improving outcomes.^1)^ With the widespread adoption of endovascular aneurysm repair (EVAR), treatment strategies for rAAA have evolved substantially. Contemporary studies have suggested that EVAR may offer perioperative survival benefits and reduced physiological stress compared with open surgical repair in appropriately selected patients.^1,2)^

The feasibility and durability of EVAR depend strongly on anatomical suitability, particularly adherence to device-specific instructions for use (IFU), which are designed to ensure adequate proximal and distal sealing.^3–5)^ In emergency settings, however, immediate hemorrhage control takes priority, and EVAR may be performed even when anatomical criteria are not met. The clinical impact of such IFU deviations on early and mid-term outcomes remains incompletely understood, especially in real-world single-center practice.

Another important aspect of rAAA management is the optimization of preoperative decision-making. In geographically large medical regions, long transfer distances can delay imaging review, device selection, and operating room (OR) preparation. Remote medical image-sharing applications, such as JOIN (Allm, Tokyo, Japan), enable prehospital transmission of computed tomography (CT) images and allow vascular surgeons to assess anatomical suitability before patient arrival.^6,7)^ Early access to contrast-enhanced CT may facilitate timely device selection and rapid intervention, particularly when hostile neck anatomy may influence the choice between EVAR and open repair.

The aim of this study was to retrospectively evaluate patients who underwent EVAR for rAAA at our institution, to compare outcomes between patients treated inside and outside IFU, and to assess the potential impact of a prehospital image-sharing application on transfer-related treatment time.

## Materials and Methods

### Study design and patient population

This single-center, retrospective observational study included consecutive patients who underwent emergency EVAR for rAAA at Asahikawa Medical University Hospital between August 2016 and June 2023. A total of 31 patients treated with emergency EVAR for rAAA were analyzed. During the study period, there was 1 case in which the patient underwent open surgery for rAAA extending from just below the renal artery bifurcation; this case was excluded. Asahikawa Medical University Hospital covers an extensive medical service area in northern Hokkaido, receiving emergency referrals from distant regions up to approximately 300 km away. To facilitate rapid prehospital evaluation, we collaborate with regional core hospitals using a secure inter-hospital communication platform (JOIN; Allm). JOIN enables real-time sharing of DICOM-based CT images obtained at referring hospitals, allowing our team to review imaging findings and prepare for emergency EVAR before patient arrival.

This study was approved by the Institutional Review Board of Asahikawa Medical University (approval number: C24085). Owing to the retrospective design, the requirement for individual informed consent was waived, and an opt-out option was provided.

### Definition of rAAA

rAAA was defined as an abdominal aortic aneurysm identified on CT with retroperitoneal hematoma, contrast extravasation, or other imaging findings suggestive of rupture, together with at least one of the following clinical features: sudden abdominal pain, back pain, or hypotension.

### Treatment strategy for rAAA

Our institution has consistently adopted an EVAR-first protocol for the management of rAAA, considering the high mortality rate of open surgery, and prioritizing rapid hemorrhage control and patient survival. EVAR was selected as the first-line treatment whenever anatomical feasibility was considered acceptable. In life-threatening situations, EVAR was also performed outside the device-specific IFU when the attending vascular surgeon judged that EVAR was technically achievable and offered the best chance of survival. The final treatment strategy was determined based on preoperative CT findings, hemodynamic status, transfer conditions, institutional resources, and device availability.

Patients with unstable hemodynamics were prioritized for immediate transfer to the OR, and intra-aortic balloon occlusion (IABO) was used when necessary to obtain proximal control. In patients with relatively stable hemodynamics, detailed CT assessment was performed to determine device selection and operative planning. When only non-contrast CT was available from the referring hospital and anatomical suitability remained uncertain, contrast-enhanced CT was obtained upon arrival before proceeding to the OR, provided that vital signs permitted.

For transferred patients, initial management followed a standardized protocol. Systolic blood pressure was maintained at approximately 70–90 mmHg to limit ongoing hemorrhage; persistent hypertension above 90 mmHg prompted initiation of continuous antihypertensive agents, whereas hypotension below 70 mmHg was managed with cautious fluid resuscitation. Maintenance of body temperature and peripheral perfusion was emphasized to prevent coagulopathy and acidosis. Analgesia was provided as needed, while sedative agents were avoided whenever possible. Respiratory support was initiated in cases of impaired consciousness or agitation, although endotracheal intubation before proximal control was avoided when possible to reduce the risk of hemodynamic deterioration associated with induction and positive-pressure ventilation. In the event of cardiac arrest during transfer, surgical intervention was not pursued unless spontaneous circulation returned.

### Perioperative management

When contrast-enhanced CT images were shared in advance through the JOIN platform and EVAR was deemed feasible, patients were transferred directly to the OR to minimize the risk of re-rupture. To prevent hemodynamic collapse during induction, IABO was first inserted via the femoral artery under local anesthesia, followed by induction of general anesthesia. In selected patients with limited hematoma, stable hemodynamics, and adequate cooperation, EVAR was performed under local anesthesia alone.

After EVAR, conversion to open surgery was considered when abdominal distension became pronounced, shock persisted, lactate levels were markedly elevated, or transfusion requirements exceeded approximately 2500 mL. To standardize postoperative decision-making, open abdominal management (OAM) was performed based on a 4-item institutional scoring system: total transfusion volume ≥2500 mL, peak intraoperative lactate levels ≥70 mg/dL, bladder pressure ≥25 mmHg, and marked abdominal distension. Patients scoring ≥2 points were considered candidates for OAM.^8)^ Because rapid stent-graft deployment to control hemorrhagic shock was prioritized, prophylactic (intraoperative) embolization of the inferior mesenteric and lumbar arteries was not routinely performed. Type II endoleaks without sac enlargement during follow-up were managed conservatively with imaging surveillance.

### Device selection

Device selection followed a standardized institutional algorithm based on proximal neck length and the presence of common iliac artery aneurysms. The devices used were Endurant (Medtronic, Minneapolis, MN, USA) and Excluder (W. L. Gore & Associates, Flagstaff, AZ, USA).

For patients with a proximal neck length ≥15 mm, Excluder was selected when common iliac artery aneurysms were present, whereas either Endurant or Excluder could be used when the iliac arteries were normal. For patients with a proximal neck length <15 mm, Endurant was preferentially selected. In cases with a short proximal neck and concomitant common iliac artery aneurysms, a chimeric configuration consisting of an Endurant main body combined with Excluder iliac limbs was used when anatomically appropriate. In this cohort, all 10 cases assigned to the Endurant group (32.3%) used this chimeric configuration, evenly distributed between the inside-IFU and outside-IFU groups (5 and 5, respectively).

In contrast, classification as inside or outside IFU—based on the complete device-specific IFU criteria (proximal neck diameter and length, infrarenal neck angulation, neck thrombus/calcification, and the iliac landing zone)—was determined retrospectively for analysis only and was used neither to determine EVAR eligibility (EVAR was performed even outside IFU) nor to guide device selection. IFU criteria were applied in a device-specific manner, using the IFU of the GORE EXCLUDER AAA Endoprosthesis with C3 Delivery System and the Endurant II/IIs available during the study period.

### Image-sharing application and transfer-related factors

In patients transferred from hospitals participating in the JOIN network, JOIN was used to securely share clinical information and imaging data.^7)^ Whether image sharing was performed was determined by the referring hospital’s participation in the network rather than by individual patient characteristics. Image sharing was performed only after obtaining patient or family consent. Shared information was simultaneously accessible to anesthesiologists, emergency physicians, and nursing staff, enabling early OR preparation and anesthesia planning. Patients were classified according to the use or nonuse of JOIN.

Transfer-related variables included transfer distance, mode of transfer, repeat CT after arrival, time from arrival to anesthesia induction, time from arrival to initial transfusion, door-to-OR time, OR-to-incision time, and total operation time. Because the timing of OR preparation can be subjective, OR activation was operationally defined as successful entry into the OR within 30 min of hospital arrival. The mode of transfer was classified as ambulance or helicopter.

### Perioperative workflow variables

Perioperative workflow variables were evaluated to assess the impact of JOIN on preoperative coordination. These included pre-arrival OR activation, pre-arrival hybrid OR activation, hybrid OR utilization, time from arrival to anesthesia induction, time from arrival to initial transfusion, and door-to-OR time. As documentation of OR preparation timing can be subjective, OR activation was operationally defined as patient entry into the OR within 30 min of arrival, reflecting timely readiness of the surgical and anesthesia teams.

### Outcome definitions

The primary outcome was reintervention. Reintervention was defined as any additional procedure performed for aneurysm-related events after the initial EVAR. Reinterventions included treatment for endoleak, treatment for stent-graft migration, proximal cuff implantation, limb extension, embolization, and open conversion.

Secondary outcomes included 30-day mortality, overall mortality, endoleak, stent-graft migration, and open conversion. Endoleaks were classified as type I to V according to standard criteria. Dates of death were confirmed using hospital records and information obtained from referring institutions.

### Follow-up

Postoperative follow-up was conducted in the outpatient clinic. Imaging surveillance was performed using contrast-enhanced or non-contrast CT, and the schedule was adjusted according to patient condition, renal function, endoleak status, and aneurysm sac changes. The observation period was defined as the interval from the initial EVAR to the date of last confirmed survival, death, or last imaging assessment.

### Statistical analysis

Continuous variables are presented as mean ± standard deviation or median (interquartile range), as appropriate. Categorical variables are presented as counts and percentages. Between-group comparisons were performed using Student’s t-test or the Mann–Whitney U test for continuous variables and the chi-square test or Fisher’s exact test for categorical variables. For categorical variables with more than 2 categories, the Fisher–Freeman–Halton exact test was applied.

Freedom from reintervention and overall survival were estimated using the Kaplan–Meier method, and differences between groups were assessed using the log-rank test. Statistical analyses were performed using R software (R Foundation for Statistical Computing, Vienna, Austria).^9)^ A 2-sided p value <0.05 was considered statistically significant.

## Results

### Baseline characteristics and procedural details

During the study period, 31 patients who underwent EVAR for rAAA were analyzed. The mean age was 77.3 ± 9.3 years, and 29 patients (93.5%) were male. Twenty patients (64.5%) were classified as inside IFU and 11 (35.5%) as outside IFU (**Table 1**). Among the 11 patients classified as outside IFU, 6 had a severely angulated proximal neck, 4 had a short neck, and 1 had significant neck thrombus. Thus, all outside-IFU cases were attributable to hostile proximal neck morphology. There were no significant differences between the inside-IFU and outside-IFU groups in baseline comorbidities, Hardman index, Fitzgerald classification, or Rutherford classification. In contrast, outside-IFU patients had significantly shorter proximal neck length (20.6 ± 12.4 mm vs. 30.9 ± 10.5 mm, p = 0.021) and greater neck angulation (63.9° ± 31.9° vs. 29.4° ± 24.9°, p = 0.002). Maximum aneurysm diameter, device type, anesthesia type, procedure duration, and IABO use did not differ significantly between groups. Although there was no statistically significant difference, Hb levels and total transfusion volume tended to be lower in outside-IFU patients upon arrival in the OR.

**Table 1:** Baseline patient characteristics and procedural details, overall and according to IFU status

	Overall (n = 31)	Inside IFU (n = 20)	Outside IFU (n = 11)	p Value
Age (years)	77.3 ± 9.3	77.8 ± 10.8	76.4 ± 6.7	0.64
Male, n (%)	29 (93.5)	19 (95.0)	10 (90.9)	1.00
Hypertension, n (%)	28 (90.3)	17 (85.0)	11 (100)	0.535
Diabetes mellitus, n (%)	5 (16.1)	3 (15.0)	2 (18.2)	1.00
Coronary artery disease, n (%)	5 (16.1)	4 (20.0)	1 (9.1)	0.631
Cerebrovascular disease, n (%)	5 (16.1)	2 (10.0)	3 (27.3)	0.317
Peripheral artery disease, n (%)	3 (9.7)	1 (5.0)	2 (18.2)	0.281
CKD Stage 4, n (%)	12 (38.7)	7 (35.0)	5 (45.5)	0.705
COPD, n (%)	6 (19.4)	3 (15.0)	3 (27.3)	0.638
Hardman index, n (%)				
0–2	25 (80.6)	16 (80.0)	9 (81.8)	0.742
3	6 (19.4)	4 (20.0)	2 (18.2)	
Fitzgerald classification, n (%)				0.83
I	0 (0)	0 (0)	0 (0)	
II	6 (19.4)	3 (15.0)	3 (27.3)	
III	22 (71.0)	15 (75.0)	7 (63.6)	
IV	3 (9.7)	2 (10.0)	1 (9.1)	
Rutherford classification, n (%)				0.815
R1	7 (22.6)	4 (20.0)	3 (27.3)	
R2	4 (12.9)	2 (10.0)	2 (18.2)	
R3	15 (48.4)	10 (50.0)	5 (45.5)	
R4	5 (16.1)	4 (20.0)	1 (9.1)	
Maximum aneurysm diameter (mm)	71.4 ± 13.0	71.2 ± 15.0	71.7 ± 9.7	0.918
Neck length (mm)	27.3 ± 11.9	30.9 ± 10.5	20.6 ± 12.4	0.021
Neck angle (°)	40.9 ± 31.0	29.4 ± 24.9	63.9 ± 31.9	0.002
Device type				0.423
Excluder, n (%)	21 (67.7)	15 (75.0)	6 (54.5)	
Endurant, n (%)	10 (32.3)	5 (25.0)	5 (45.5)	
Anesthesia				
Local, n (%)	5 (16.1)	4 (20.0)	1 (9.1)	0.631
Local to general, n (%)	13 (41.9)	10 (50.0)	3 (27.3)	0.275
General, n (%)	13 (41.9)	6 (30.0)	7 (63.6)	0.128
Hemoglobin at OR arrival	8.97 ± 2.77	9.65 ± 2.76	7.74 ± 2.45	0.065
Total transfusion volume (mL)	2210 (0–12880)	1725 (0–9610)	3410 (1520–12880)	0.060
Total fluid volume (mL)	2200 (650–7630)	2190 (650–5200)	2250 (700–7630)	0.788
Maximum lactate value (mg/dL)	60.4 ± 39.7	57.4 ± 34.6	66.3 ± 49.9	0.572
Procedure duration (min)	209 ± 88.8	198 ± 74.9	228 ± 114.6	0.382
Door-to-anesthesia time (min)	32 (6–140)	31 (6–109)	39 (7–140)	0.772
Time from arterial access to stent-graft deployment (min)	33 (12–107)	31 (17–75)	34 (12–107)	0.918
IABO, n (%)	23 (74.2)	15 (75.0)	8 (72.7)	1.00

All 10 cases in the Endurant group were chimeric configurations (Endurant main body with Excluder iliac limbs); inside IFU, n = 5; outside IFU, n = 5.

CKD, chronic kidney disease; COPD, chronic obstructive pulmonary disease; IABO, intra-aortic balloon occlusion; IFU, instructions for use

### Reintervention and perioperative outcomes

Long-term reintervention occurred in 2 patients (10.0%) in the inside-IFU group and 7 patients (63.6%) in the outside-IFU group, with a significantly higher incidence in the outside-IFU group (p <0.01) (**Table 2**). Within 30 days, reintervention was required in 1 patient (5.0%) in the inside-IFU group and 2 patients (18.2%) in the outside-IFU group (p = 0.234). Two of these 3 early reinterventions were for type Ia endoleak (1 with a concomitant type Ib component treated with iliac limb extension), whereas the remaining case underwent open hematoma evacuation with ligation of the lumbar and inferior mesenteric arteries for postoperative hemorrhage/type II endoleak.

**Table 2:** Clinical outcomes after endovascular aortic repair for ruptured abdominal aortic aneurysm according to IFU status

	Inside IFU (n = 20)	Outside IFU (n = 11)	p Value
Early outcome			
30-day reintervention, n (%)	1 (5.0)	2 (18.2)	0.234
Aortic cuff for type Ia endoleak	1	1	
Limb extension for type Ib endoleak	1	0	
Open hematoma evacuation and lumbar/IMA ligation for type II endoleak	0	1	
Open abdominal management, n (%)	9 (45.0)	4 (36.4)	1.000
30-day mortality, n (%)	2 (10.0)	2 (18.2)	0.601
Long-term outcome			
Endoleak, n (%)	4 (20.0)	6 (54.5)	0.132
Type I/II/III/IV/V	2/1/0/1/0	2/3/0/0/1	
Reintervention, n (%)	2 (10.0)	7 (63.6)	<0.01
Aortic cuff, n (%)	0	1 (9.1)	0.355
Limb extension, n (%)	1 (5.0)	3 (27.3)	0.115
Lumbar artery embolization, n (%)	1 (5.0)	1 (9.1)	1.000
Late open surgical conversion, n (%)	1 (5.0)	4 (36.4)	0.042
Overall outcome			
Multiple reinterventions	1 (5.0)	4 (36.4)	0.023
All-cause mortality, n (%)	12 (60.0)	4 (36.4)	0.300

IFU, instructions for use

Endoleaks occurred in 4 patients (20.0%) inside IFU and 6 patients (54.5%) outside IFU, without a statistically significant difference (p = 0.132). Late open surgical conversion was significantly more frequent in the outside-IFU group (36.4% vs. 5.0%, p = 0.042). Multiple reinterventions were also significantly more common in the outside-IFU group (36.4% vs. 5.0%, p = 0.023).

The 30-day mortality rate was 10.0% in the inside-IFU group and 18.2% in the outside-IFU group (p = 0.601). All-cause mortality was 12/20 (60.0%) in the inside-IFU group and 4/11 (36.4%) in the outside-IFU group (p = 0.300). Kaplan–Meier analysis demonstrated significantly lower freedom from reintervention in the outside-IFU group compared with the inside-IFU group (log-rank p = 0.0057) (**Fig. 1A**), whereas overall survival did not differ substantially between the groups (**Fig. 1B**).

**Fig. 1:**
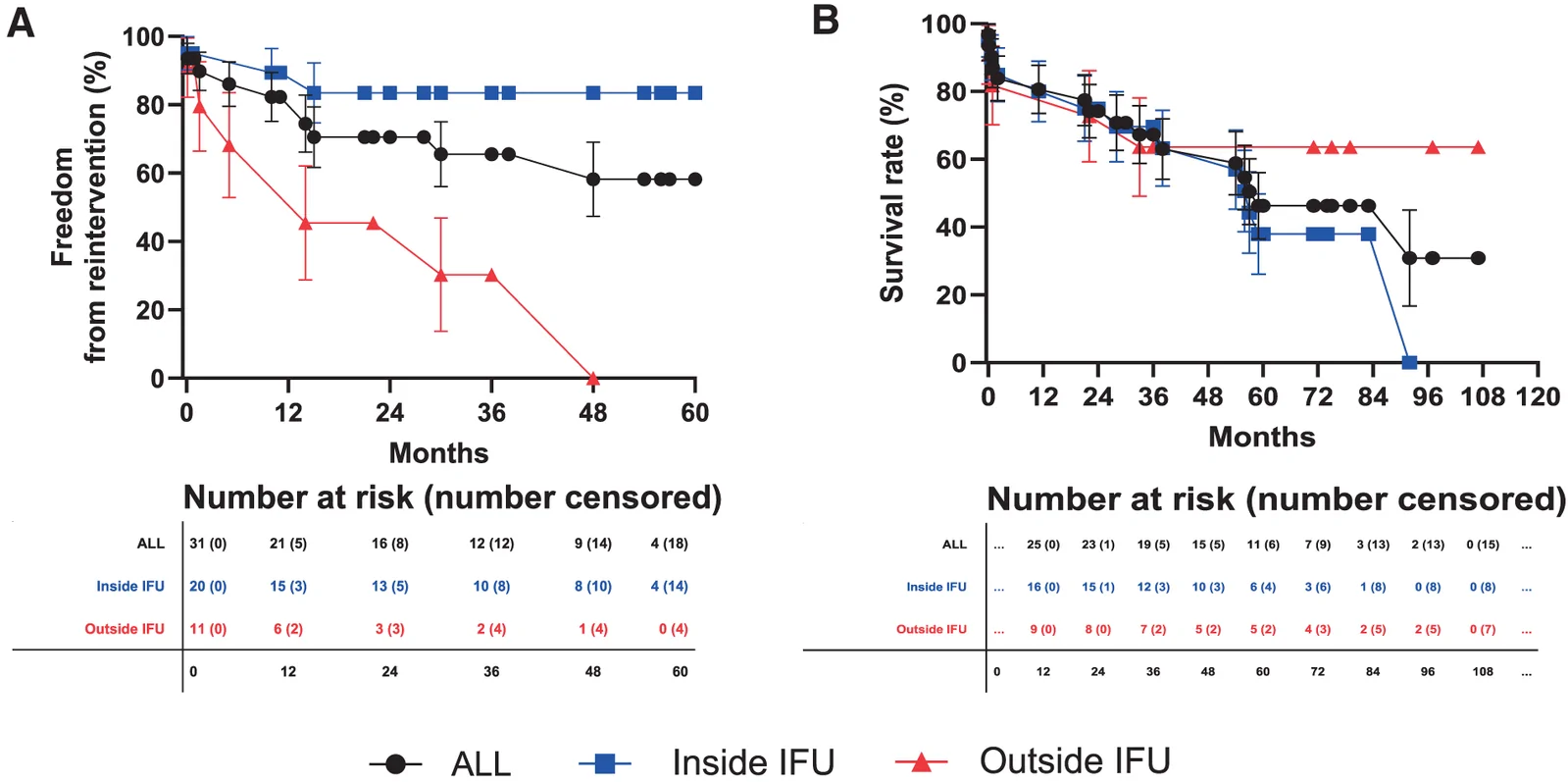
(**A**) Freedom from reintervention after EVAR according to IFU status. (**B**) Overall survival after EVAR according to IFU status. EVAR, endovascular aneurysm repair; IFU, instructions for use

### Details of reinterventions in patients outside IFU

Details of reinterventions in patients outside IFU are summarized in **Table 3**. Among patients outside IFU, 4 of the 6 patients (66.7%) with an angulated neck required reintervention. The procedures comprised attempted lumbar artery embolization followed by open surgical conversion for a type II endoleak, limb extension for distal limb migration, open hematoma evacuation with lumbar artery ligation followed by aortic cuff placement and limb extension for proximal main-body migration, and open surgical conversion for an internal iliac artery aneurysm. One patient with an angulated neck experienced early mortality due to advanced cancer. Among the 4 patients with a short neck, 3 (75.0%) required reintervention, comprising aortic cuff placement for a type Ia endoleak, open surgical conversion for proximal main-body migration, and limb extension with open surgical conversion for a type Ia endoleak. One patient with a short neck experienced early mortality due to type Ia endoleak. No reintervention occurred in the patient with neck thrombus. Reintervention occurred predominantly among patients with hostile neck morphology, including an angulated neck (4/6) and a short neck (3/4).

**Table 3:** Patient-level summary of the 7 patients treated outside the IFU who underwent reintervention

Case	Reason for outside IFU	Indication for reintervention	Timing	Reintervention procedure
1	Angulated neck	Type II endoleak	Late	Attempted lumbar artery embolization via the right internal iliac artery, followed by open surgical conversion
2	Angulated neck	Limb migration	Late	Limb extension from the common to the external iliac artery
3	Angulated neck	In-stent-graft thrombosis	Late	Open surgical conversion
4	Angulated neck	Type Ia endoleak with proximal migration	Early	Aortic cuff placement
5	Short neck	Postoperative hemorrhage/type II endoleak	Early	Open hematoma evacuation with closure of the rupture site and ligation of the lumbar and inferior mesenteric arteries
5	Short neck	Main-body migration	Late	Aortic cuff placement and limb extension
6	Short neck	Main-body migration	Late	Open surgical conversion
7	Short neck	Sac re-expansion with suspected type Ia endoleak	Late	Emergency laparotomy with limb extension, subsequent open surgical conversion, and further limb extension

Early, ≤30 days after the initial endovascular aneurysm repair; late, >30 days.

Case 5 underwent reintervention at 2 separate time points and is therefore listed twice; Cases 1 and 7 underwent more than 1 procedure within a single reintervention. Procedure-level counts in **Table 2** therefore exceed the number of patients.

Reintervention occurred in 4 of the 6 patients with an angulated neck (66.7%) and in 3 of the 4 patients with a short neck (75.0%). The 4 outside-IFU patients who did not undergo reintervention were the single patient classified outside IFU because of neck thrombus (alive), 2 patients who died within 30 days (1 with an angulated neck, from advanced cancer; 1 with a short neck, from type Ia endoleak with re-rupture), and 1 patient with an angulated neck who died beyond 30 days.

IFU, instructions for use

### JOIN use and transfer-related treatment time

JOIN was used in 12 patients and not used in 19 patients. Patient characteristics and clinical/hemodynamic severity (including shock vital signs, Hardman index, and Fitzgerald and Rutherford classifications) did not differ significantly between the 2 groups. Patients in the JOIN group had significantly longer transfer distances than those in the non-JOIN group [80 (5.4–122) km vs. 5.5 (1.5–324) km, p = 0.002] (**Table 4**). Despite the longer transfer distance, preoperative processes were markedly more efficient in the JOIN group. Preoperative CT sharing was achieved in all patients in the JOIN group, and repeat CT after arrival was significantly less frequent (8.3% vs. 47.4%, p = 0.023). Pre-arrival OR activation was substantially more common in the JOIN group (91.7% vs. 31.6%, p <0.01), as was pre-arrival hybrid OR activation (91.7% vs. 26.3%, p <0.01). Hybrid OR utilization was high in both groups.

**Table 4:** Transfer-related variables and outcomes according to JOIN use

	Overall (n = 31)	JOIN (+) (n = 12)	JOIN (−) (n = 19)	p Value
Transfer distance (km)	22 (1.5–324)	80 (5.4–122)	5.5 (1.5–324)	0.002
Arrival method				
Ambulance, n (%)	20 (64.5)	6 (50.0)	14 (73.7)	0.088
Helicopter, n (%)	9 (29.0)	6 (50.0)	3 (15.8)	
Walk-in, n (%)	2 (6.5)	0 (0)	2 (10.5)	
Preoperative CT evaluation	31 (100)	12 (100)	19 (100)	
No contrast-enhanced CT before operation	11 (35.5)	5 (41.7)	6 (31.6)	0.642
Contrast-enhanced CT	20 (64.5)	7 (58.3)	13 (68.4)	
Repeat CT after arrival, n	10 (32.3)	1 (8.3)	9 (47.4)	0.023
Outside IFU, n (%)	11 (35.5)	1 (8.3)	10 (52.6)	0.020
Pre-arrival OR activation, n (%)	17 (54.8)	11 (91.7)	6 (31.6)	<0.01
Pre-arrival hybrid OR activation, n (%)	16 (51.6)	11 (91.7)	5 (26.3)	<0.01
Hybrid OR utilization, n (%)	28 (90.3)	12 (100)	16 (84.2)	0.265
Door-to-OR time, min	17 (2–130)	13.5 (6–34)	64 (2–130)	0.026
Time from arrival to anesthesia induction, min	32 (6–140)	17.5 (7–37)	66 (6–140)	0.030
Lactate on arrival, mg/dL	29 (8–114)	27.5 (8–85)	29 (10–114)	0.855
Hemoglobin at OR arrival (g/dL)	8.97 ± 2.77	8.72 ± 2.89	9.13 ± 2.76	0.69
OR-to-incision time, min	17 (1–37)	18.5 (1–37)	14 (5–30)	0.503
Time from arrival to initial transfusion, min	113 (13–251)	99.5 (13–251)	119 (37–177)	0.037
Anesthesia time, min	257 (107–530)	229 (124–343)	257 (107–530)	0.570
Operation duration, min	209 ± 90.3	192 ± 76.3	221 ± 98.4	0.393
Total transfusion volume, mL	2210 (0–12880)	1485 (0–3410)	2640 (280–12880)	0.059
Total fluid volume, mL	2200 (650–7630)	1775 (700–7630)	2550 (650–5200)	0.133
IABO, n (%)	23 (74.2)	9 (75.0)	14 (73.4)	1.000
Maximum lactate level, mg/dL	60.4 ± 39.7	59.3 ± 35.5	61.1 ± 43.3	0.910
OAM, n (%)	13 (41.9)	4 (33.3)	9 (47.3)	0.465
30-day mortality, n(%)	4 (12.9)	0 (0)	4 (21.1)	0.139
Overall mortality, n (%)	16 (51.6)	7 (58.3)	9 (47.4)	0.716

CT, computed tomography; OR, operating room; IABO, intra-aortic balloon occlusion; OAM, open abdominal management; IFU, instructions for use

Time from arrival to anesthesia induction was significantly shorter in the JOIN group [17.5 (7–37) min vs. 66 (6–140) min, p = 0.030], indicating that JOIN facilitated rapid anesthetic preparation and decision-making. Importantly, the time from arrival to initial transfusion was also significantly shorter [99.5 (13–251) min vs. 119 (37–177) min, p = 0.037], reflecting earlier initiation of hemostatic resuscitation. Consistent with these improvements in preoperative workflow, door-to-OR time was markedly shorter in the JOIN group [13.5 (6–34) min vs. 64 (2–130) min, p = 0.026]. In contrast, OR-to-incision time, anesthesia time, and operation duration did not significantly differ between groups, suggesting that JOIN primarily influenced pre-OR workflow rather than intraoperative processes. Lactate levels on arrival were comparable between groups, indicating similar physiological severity at presentation. Total transfusion volume tended to be lower in the JOIN group [1485 (0–3410) mL vs. 2640 (280–12880) mL, p = 0.059], although the difference was not statistically significant, whereas total fluid volume did not differ significantly. Thirty-day mortality and overall mortality did not differ significantly between the JOIN and non-JOIN groups.

## Discussion

In this single-center retrospective study of 31 patients who underwent EVAR for rAAA, 35.5% were treated outside the device IFU, and all outside-IFU cases were attributable to hostile proximal neck anatomy. Outside-IFU EVAR was associated with a significantly higher reintervention rate, whereas 30-day and overall mortality did not differ significantly between the inside-IFU and outside-IFU groups. These findings indicate that EVAR can provide acceptable early survival even in anatomically challenging rAAA cases, although unfavorable neck anatomy remains a key determinant of long-term device durability and the need for secondary intervention.

A second major finding of this study was the substantial improvement in preoperative workflow associated with the use of a real-time image-sharing platform (JOIN). Despite significantly longer transfer distances, patients in the JOIN group experienced markedly shorter times from arrival to anesthesia induction, earlier initiation of transfusion, and significantly reduced door-to-OR time. These improvements likely reflect the ability of JOIN to facilitate pre-arrival CT sharing, early OR preparation, and coordinated readiness among surgical, anesthesia, and nursing teams. Although mortality did not differ significantly according to JOIN use, the reductions in transfusion requirements and acceleration of preoperative processes suggest that JOIN may contribute to more efficient hemorrhage control and stabilization during the acute phase of rAAA management. Because lactate and hemoglobin levels at OR arrival were comparable between the groups, the accompanying trend toward lower transfusion volume more plausibly reflects the shortened workflow and earlier hemostasis than baseline differences, although it did not reach significance in a small retrospective sample.

At our institution, OAM was a proactive, planned strategy to prevent abdominal compartment syndrome rather than a complication, which accounts for the relatively high early OAM rate. Despite a longer transfer distance (JOIN 80 vs. 5.5 km, p = 0.002), the JOIN group had a lower OAM rate (33.3% vs. 47.3%, p = 0.465), suggesting that JOIN may offset part of the disadvantage of long-distance transfer. Because onset-to-OR time was not recorded and the mortality comparison is underpowered, JOIN is best regarded as a workflow advantage rather than a survival benefit.

Contemporary practice for rAAA has increasingly shifted toward an EVAR-first strategy. Varkevisser et al. reported that 5-year survival after EVAR for rAAA has improved over time, whereas survival after open repair has remained relatively constant.^1)^ The IMPROVE trial also demonstrated that an endovascular strategy can be applied as an emergency treatment pathway for suspected rAAA, with advantages in selected outcomes and resource use.^2)^ Our institution has similarly adopted an EVAR-first protocol incorporating rapid CT assessment, local anesthesia when feasible, and IABO for hemodynamic control. The absence of a significant survival difference between the inside-IFU and outside-IFU groups in the present study may reflect the value of this streamlined approach in the acute phase.

However, the durability of EVAR performed outside IFU remains a major concern. In the present study, the leading causes of reintervention—type Ia endoleak, proximal and distal migration, and persistent type II endoleak requiring open conversion—were anatomically consistent with the fact that all outside-IFU cases had hostile proximal neck morphology. The association between unfavorable neck anatomy and subsequent device-related complications has been repeatedly demonstrated.^3)^ Short proximal neck has been strongly linked to type Ia endoleak,^4)^ and Endurant implantation outside the proximal neck IFU has shown inferior medium- and long-term durability.^5)^ An increased risk of neck-related adverse events has also been observed when EVAR is performed outside the proximal neck IFU, irrespective of rupture status.^10)^ Taken together, these findings reinforce that anatomical deviation—particularly hostile proximal neck anatomy—is not merely a technical challenge at the index procedure but a fundamental determinant of later aneurysm-related events. Because these comparisons were unadjusted for confounders, outside-IFU anatomy should be interpreted as associated with, rather than as an independent predictor of, reintervention; notably, only 2 of the 9 reinterventions occurred in chimeric cases (evenly split between groups), so the excess reintervention is unlikely to reflect the chimeric configuration. Despite no routine embolization of the inferior mesenteric or lumbar arteries, type II endoleak and secondary intervention rates remained low—possibly reflecting frequent side-branch thrombosis in ruptured cases and conservative management—though this should be interpreted cautiously given the small sample.

A further observation emerges from the retrospective design itself. Our device-selection algorithm was keyed to proximal neck length and the presence of common iliac artery aneurysm; no patient had a proximal neck length falling between the minimum neck-length requirements of the 2 devices, so device choice could not have altered IFU classification. Infrarenal neck angulation, by contrast, was not a selection variable—under an EVAR-first protocol it was accepted as a known risk. Yet reintervention was as frequent among patients with an angulated neck (4/6) as among those with a short neck (3/4), and angulation separated the IFU groups more strongly than neck length. Although the small sample precludes formal comparison between individual anatomical features, the feature that did not inform our decisions appears to have been as consequential as the one that did. Explicit assessment of angulation is therefore a necessary complement to an EVAR-first policy rather than an argument against it.

The clinical question is not simply whether EVAR outside IFU is acceptable, but when it is justified. In rAAA, profound hemodynamic instability often leaves little time for detailed deliberation, and immediate hemorrhage control may take precedence over ideal anatomical suitability. For elderly or high-risk patients, EVAR may be the only realistic option to achieve early survival.^11)^ Hoshina et al. reported favorable nationwide outcomes of EVAR for abdominal aortic aneurysm in Japan, supporting its broad use in contemporary practice.^12)^ Nevertheless, outside-IFU EVAR for rAAA should be considered a deliberate, patient-specific strategy rather than a routine expansion of indications. In some cases, EVAR may function as a bridging therapy to achieve survival in the acute phase, followed by planned surveillance, secondary endovascular repair, or open conversion when necessary.^13)^ Late open surgical conversion was significantly more frequent in the outside-IFU group (36.4% vs. 5.0%, p = 0.042), consistent with the hostile proximal neck anatomy of all outside-IFU cases and typically triggered by type Ia endoleak or proximal main-body migration. Although outside-IFU EVAR is valuable as acute-phase, life-saving hemostasis, it carries a substantial likelihood of secondary intervention, including open conversion, over the long term, reinforcing the need for careful case selection, strict imaging surveillance, and preparedness for secondary intervention.

These considerations inevitably influence the choice between EVAR and open surgical repair. Outside-IFU anatomy—particularly short or severely angulated proximal neck—should prompt explicit consideration of open repair when the patient’s physiological reserve and institutional resources permit.^14)^ Conversely, in patients with shock, advanced age, or prohibitive operative risk, EVAR may remain appropriate despite anatomical disadvantages, provided that the higher likelihood of reintervention is recognized and incorporated into postoperative planning. Thus, treatment selection should integrate hemodynamic status, age, comorbidities, anatomical risk, device availability, and the feasibility of structured follow-up.

Substantial variation in hospital door-to-intervention time for rAAA has been documented in contemporary practice, underscoring the persistent difficulty in achieving timely operative access.^15)^ In our cohort, prehospital image sharing was associated with shorter door-to-OR time despite markedly longer transfer distance, indicating that pre-arrival image review can facilitate earlier anatomical assessment, device selection, team activation, and OR preparation. Recent evidence highlights the importance of prehospital assessment in rAAA management, particularly for rapid triage and early treatment planning.^16)^ Although diagnostic delay or initial misdiagnosis has not always been shown to worsen operative outcomes in rAAA,^17)^ EVAR specifically requires accurate anatomical evaluation and early sharing of imaging information—especially in patients with hostile neck anatomy, where detailed assessment of neck morphology and access vessels is essential for anticipating procedural complexity. In geographically dispersed regions, rapid stabilization of hemodynamic status during transfer is also critical for survival, as demonstrated in studies of acute aortic emergencies and contemporary rAAA cohorts showing the prognostic impact of prehospital hemodynamics.^18,19)^ Taken together, these findings suggest that image-sharing systems can serve as a practical method to reduce in-hospital delay, support appropriate treatment selection, and enhance early stabilization across wide-area emergency networks. The JOIN network currently lacks a formal protocol for contrast-enhanced computed tomography angiography (CTA) at referring institutions, and 38.7% of cases had only non-contrast CT, largely reflecting limited pre-transfer time in unstable rAAA; disseminating imaging protocols for hemodynamically tolerant cases is a future goal. As JOIN is used primarily on smartphones, its role lies in rapid morphological assessment and the EVAR-versus-open decision rather than workstation-level sizing, and a PC-based version could further improve pre-transfer assessment. Incorporating morphological assessment beyond a rapid feasibility judgment—in particular proximal neck angulation—into the pre-arrival review could extend its contribution from how EVAR is performed to whether EVAR or open repair is the appropriate operation.

This study has several limitations. First, it was a single-center retrospective analysis with a small sample size, which limits statistical power and increases the risk of type II error. In addition, owing to the small sample size, no multivariable (e.g., Cox) regression analysis was performed; the reported outcomes are therefore unadjusted for potential confounders such as maximum aneurysm diameter and iliac anatomy. IFU status should accordingly be regarded as a descriptive, hypothesis-generating marker rather than an independent predictor of reintervention. Second, patients who underwent open repair as the initial strategy were not included, preventing direct comparison between EVAR and open repair. Third, IFU suitability was assessed retrospectively and may not fully reflect real-time decision-making in emergency settings. Accordingly, although outside-IFU treatment was less frequent in the JOIN group, the small number of patients precludes any conclusion that image sharing improved IFU compliance. Finally, follow-up intervals and imaging modalities were not fully standardized, which could have influenced the detection of endoleak and other aneurysm-related events. In addition, the time from symptom onset/rupture to OR arrival was not recorded, precluding direct assessment of its influence on the OAM rate and on outcomes; consequently, the longer transfer distance in the JOIN group could not be accounted for, and a mortality benefit of JOIN cannot be directly assessed. Despite these limitations, the study provides real-world insight into the balance between early survival and later reintervention after outside-IFU EVAR for rAAA.

## Conclusion

EVAR for rAAA achieved acceptable early survival, but outside-IFU anatomy—particularly hostile proximal necks—was associated with substantially higher reintervention. EVAR outside IFU should therefore be limited to carefully selected patients with strict postoperative surveillance and readiness for secondary intervention. Prehospital image sharing shortened door-to-OR time and supported rapid diagnosis and treatment-strategy decision; incorporating morphological assessment—particularly proximal neck angulation—into pre-arrival review may extend its contribution to the choice between endovascular and open repair.

## Declarations

### Data availability

The data used for this case report are available from the corresponding author upon request.
